# Fungaemia caused by obstructive renal candida bezoars leads to bilateral chorioretinitis: a case report

**DOI:** 10.1186/s12894-018-0335-6

**Published:** 2018-03-16

**Authors:** Johannes Stein, Stefan Latz, Jörg Ellinger, Guido Fechner, Maher Safi, Philipp Krausewitz, Simone Müller, Karin Weyer, Stefan C. Müller

**Affiliations:** 1 Department of Urology, University of Bonn, Sigmund-Freud-Str. 25, D-53127 Bonn, Germany; 2 Department of Ophthalmology, University of Bonn, Sigmund-Freud-Str. 25, D-53127 Bonn, Germany

**Keywords:** Candida chorioretinitis, Candida glabrata, Fungaemia, Obstructive nephropathy, Renal bezoars

## Abstract

**Background:**

Renal fungal bezoars are remarkably rare and mostly occur in immunodeficient patients. Only a small number of cases with immunocompetent patients have been published so far. The published treatment approaches comprised systemic antimycotic therapy and surgical or minimal invasive removal of the fungal balls. In some cases irrigation of the renal duct system with amphotericin B was performed.

By obstruction of the urinary tract bezoars can lead to infected hydronephrosis and severe urosepsis with high lethality. Fungaemia can cause fungal colonization in different distant organs. Fulminant chorioretinitis and irreversible visual impairment can be the consequence of ocular fundus colonization. The following report highlights that a co-operation between urologists and ophthalmologists is absolutely indispensible in case of fungaemia.

**Case presentation:**

Hereinafter we describe a case of an immunocompetent 56 years old woman, presenting with flank pain and shivering. The diagnosis turned out to be difficult due to initially negative urine culture. The fungaemia caused by obstructive nephropathy led to bilateral candida chorioretinitis. The patient was treated with intravenous amphotericin b and the bezoar was removed by percutaneous “nephrolitholapaxy”.

After two months, a follow up revealed the patient felt well, chorioretinal lesions regressed and urine culture did not show any fungal growth.

**Conclusion:**

To the best of our knowledge, this is the first case reporting on obstructive renal bezoars, which lead to haematogenous fungus spread and bilateral chorioretinitis.

It points out that extensive ophthalmologic examination should be performed in case of fungaemia even if the patient is not suffering from any visual impairment.

## Background

Renal candida bezoars are remarkably rare and mostly occur in immunodeficient patients. As bezoars can easily be misdiagnosed as urolithiasis a delayed or wrong therapy could be the result.

By obstruction of the urinary tract renal bezoars can quickly lead to fungaemia and cause fulminant disease progression with high lethality.

Although an interdisciplinary treatment of a patient by urologists and ophthalmologists is unusual, it is indispensable in case of fungaemia.

This case description outlines particular challenges of the diagnosis of renal bezoars and provides an overview of therapy options.

## Case presentation

**Fig. 1:**
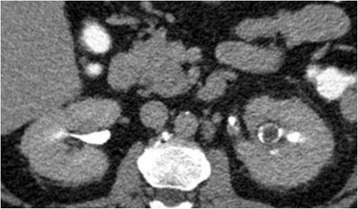
CT scan showed an obstruction of the renal pelvis caused by an amorphous mass (Hounsfield units: 13)

In February 2017, a 56-year old woman was transferred from a peripheral hospital to the department of Urology (University of Bonn) due to left sided flank pain and elevated serum infection parameters. An abdominal computed tomography scan showed an obstruction of the renal pelvis caused by an amorphous mass (Fig. 1).

The patient had neither relevant urologic medical history nor hematuria but she reported intermittent shivering since one week. The physical examination showed severe left sided flank pain on palpation. Extensive laboratory examination revealed leucocytosis (22.53 G/l), elevated C-reactive protein (253 mg/dl) and creatinin (1.4 mg/dl) as well as significant leucocyturia. A calculated antibiotic therapy with ceftriaxone and tobramycin was initiated and a double-J-stent was inserted into the left ureter. The urine culture of the admission day did not reveal bacterial growth.

Due to missing clinical recovery with persistent fever and elevated serum infection parameters, the antibiotic treatment was changed to meropenem after four days.

Eventually, repeated urine culture and blood cultures revealed fungaemia with Candida glabrata. Therefore, intravenous antifungal therapy with caspofungin was started. Due to an allergic reaction with exanthema the therapy was shifted to amphotericin B. Blood tests ruled out HIV infection and Diabetes and there was no evidence for other immune deficiencies.

**Fig. 2:**
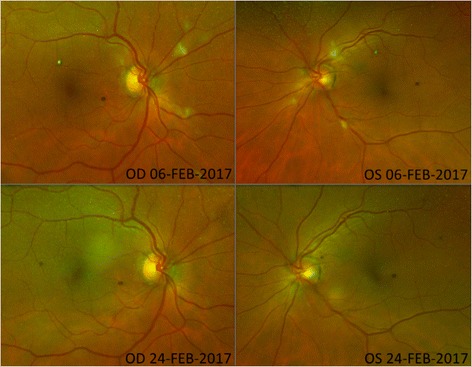
Funduscopic findings in right eye (OD) and left eye (OS) before (upper line) and after (lower line) initiation of systemic antimycotic treatment. Well demarcated parapapillary retinal infiltrates regress under antimycotic treatment

Cerebral and thoracoabdominal computed tomography scan excluded an extrarenal focus of infection and echocardiography did not show any intracardial fungal vegetations. Although the patient did not suffer from any visual impairment we initiated ophthalmoscopic examination as recommended in literature in case of fungaemia [1]. Funduscopy revealed fungal parapapillary chorioretinal infiltrates (Fig. 2). Under the antimycotic treatment, the inflammatory parameters were regressive and the patient’s clinical condition improved significantly. Ureterorenoscopic examination revealed a tough yellowish-gray mass in the renal pelvis.

**Fig. 3:**
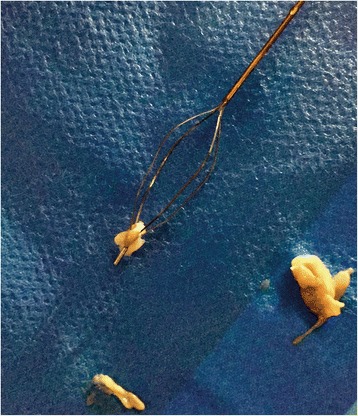
Parts of the fungal bezoars after removal

Due to insufficient ureterorenoscopic removal of the mass, it was decided to perform percutaneous “nephrolitholapaxy”. Thus the material could be extracted completely (Fig. 3). Microbiological and histological work-up revealed fungal bezoar colonized with Candida glabrata. Seven days after intervention urine culture control proved the absence of fungal colonization.

Finally, after 22 days of amphotericin B therapy, ophthalmologic re-examination showed a complete regression of the chorioretinal candida infiltrates and the patient was discharged in good general condition with normalized infection parameters.

After a follow up of two months the patient felt well and urine culture was sterile.

## Discussion

Although yeast infections of the urinary tract are common findings, obstructive uropathy caused by fungal bezoars is exceptionally rare and mostly occur in patients with diabetes or immunosuppression [2–6]. Only a small number of cases with immunocompetent patients have been published so far [7]. These patients were treated with systemic antimycotic agents and by surgical or minimal invasive removal of the fungal balls. In some cases irrigation of the renal duct system with amphotericin B was performed [2, 6, 8]. As our case has shown, it is difficult to remove the viscous material by ureterorenoscopy. It has to be discussed whether an initial percutaneous nephrostomy or an internal DJ stent is preferable in these cases. Arguments for a percutaneous nephrostomy are the direct drainage of the urine and the pre-existing access for the subsequent removal of the bezoars. However, a pre-interventional diagnosis is challenging as our case highlights.

## Conclusion

To the best of our knowledge, this is the first case reporting on obstructive renal bezoars, which lead to haematogenous spread and bilateral fungus colonization of the ocular fundus.

It points out that extensive ophthalmologic examination should be performed in case of fungaemia even if the patient is not suffering from any visual impairment. Antimycotic intravenous therapy should be applied until ocular fungal infiltrates have completely disappeared.
